# Xantogranulomatous pyeloneprhritis in children

**DOI:** 10.1007/s13244-018-0631-4

**Published:** 2018-05-23

**Authors:** Cinta Sangüesa Nebot, Sara Picó Aliaga, Agustín Serrano Durbá, María José Roca

**Affiliations:** 1 Radiology Department, Hospital Universitario y Politécnico La Fe Pediatric Imaging Section, Avenida Fernando Abril Martorell 106, 46026 Valencia, Spain; 2 Pediatric Urology Department, Hospital Universitario y Politécnico La Fe Pediatric Imaging Section, Avenida Fernando Abril Martorell 106, 46026 Valencia, Spain; 3 Pathology Department, Hospital Universitario y Politécnico La Fe Pediatric Imaging Section, Avenida Fernando Abril Martorell 106, 46026 Valencia, Spain

**Keywords:** Xanthogranulomatous, Pyelonephritis, Urolithiasis, Ultrasonography, Computed tomography

## Abstract

**Abstract:**

Xanthogranulomatous pyelonephritis (XPN) is an unusual and severe form of chronic inflammatory lesion of the kidney, characterised by the destruction of the renal parenchyma and the presence of multinucleated giant cells and lipid-laden macrophages, inflammatory infiltration and intensive renal fibrosis. There are a few cases in the literature which describe the disease in children. The pathomechanism of XPN is poorly understood. Renal obstruction with concomitant urinary tract infection is the most commonly associated pathological finding. The process is typically unilateral and may be focal or diffuse. In both cases, the perirenal infiltration is possible and can be mistaken for common renal neoplasm or inflammatory process. The symptoms are non-specific. Diagnostic imaging techniques with clinical suspicion have enabled XPN to be diagnosed and differentiated from malignancy with a high degree of confidence. Computed tomography (CT) is the mainstay of diagnostic imaging. The definitive diagnosis of XPN is based on pathological assessment after nephrectomy. We review and illustrate the clinical, radiological, surgical and pathological characteristics of XPN in children. All cases shown are surgically and histopathologically proven.

**Teaching Points:**

*• XPN can present different clinical manifestations.*

*• CT is the mainstay of diagnostic imaging in XPN.*

*• Focal type of XPN should be included in the differential diagnosis of children with a renal mass.*

*• There are no clear guidelines on the management of XPN.*

*• Conservative and surgical treatments should be considered for each individual case.*

*• Histopathological examination confirms the diagnosis and excludes other benign and malign diseases.*

## Introduction

Xanthogranulomatous pyelonephritis (XPN) is an unusual, atypical and severe form of chronic inflammatory lesion of the kidney, characterised by the destruction of the renal parenchyma and the presence of multinucleated giant cells and lipid-laden macrophages, as well as inflammatory infiltration and intensive renal fibrosis.

XPN is predominantly a disease of adults. In children it is diagnosed sporadically and is extremely rare in infants. The age of onset varies (21 days to 16 years), although 60–75% of cases have been diagnosed before 5 years of age [1]. It affects boys and girls equally, and it is slightly more common in the left kidney. The pathomechanism of XPN is poorly understood. Some factors have been implicated, chronic urinary obstruction, ineffectively treated chronic infection by *Proteus* spp., *Escherichia coli*, *Pseudomonas* spp. and *Klebsiella* spp., lipid metabolism disorders and altered immune response [2].

Congenital urinary tract malformations are highly linked with development of XPN in children (vesico–ureteral reflux, ureteropelvic junction obstruction, extrophia vesical).

Obstruction due to renal calculi has been reported in 38–83% of cases and 31–50% of the calculi have been of the staghorn variety [3].

Since the youngest patient described was a baby boy aged 21 days [4], other factors have also been implicated in the aetiology and include a lymphatic obstruction, malnutrition, arterial insufficiency, venous occlusion and haemorrhage, and necrosis of pericaliceal fat [2].

Two forms of XPN have been described: a diffuse form (which occurs in about 75% or 90% of cases) [5, 6] and a focal (or pseudo-tumoural) form, which is less frequent and more commonly described in children.

The diffuse process has characteristic imaging features. The involved kidney enlarges but maintains its reniform shape; it shows nephrolithiasis, hydronephrosis, and loss and replacement of the normal corticomedullary junction by yellow masses extending to the renal pelvis and perirenal fat.

The focal form disease is confined to a renal segment or one pole of a duplex system, and the most common location is the lower pole of the kidney [7].

The perirenal infiltration is possible in both types and can be mistaken for common renal neoplasm (Wilms’ tumour, clear cell carcinoma) or inflammatory process (renal or perirenal abscess, renal tuberculosis, focal or diffuse nephritis, sarcoidosis or Wegener granulomatosis disease).

Lesions are frequently unilateral. Bilateral XPN is extremely rare. Only ten cases of bilateral diffuse and six cases of bilateral focal XPN have been reported in the literature [8, 9].

The treatment of the diffuse type usually requires a total nephrectomy with or without antibiotic therapy. The prognosis after surgery is excellent, and no recurrence of XPN in the healthy contralateral kidney has yet been reported. However, routine follow-up, including clinical examination, ultrasonography (US), blood count and urine examination is recommended to monitor the recurrence of stone disease in the contralateral unit and the development of urinary tract infection.

The less common segmental type can be cured by partial nephrectomy or by first-line antibiotic therapy, with no recurrence reported in published studies [6]. The main problem is to confirm this diagnosis and exclude malign diseases. It is essential—in the presence of intrarenal mass—to perform biopsies for appropriate surgical management of focal XPN in order to avoid unnecessary total nephrectomy.

The radiological diagnosis of XPN cannot be carried out on the basis of a single test. Radiological features of US and computed tomography (CT) in combination with clinical suspicion must enable a diagnosis. Magnetic resonance imaging (MRI) is used most frequently. A Tc-99m DTPA (diethylene-triamine-penta-acetate) renal scan demonstrates non-function of the affected kidney.

The definitive diagnosis is based on pathological assessment after nephrectomy, which shows areas of parenchymal destruction and necrosis, as well as interstitial fibrosis and chronic inflammatory infiltrate composed of plasma cells, lymphocytes and numerous lipid-laden macrophages.

We review and illustrate the clinical, radiological, surgical and pathological characteristics of XPN in children. All cases shown are surgically and histopathologically proven.

## Clinical manifestations

The onset of the illness is usually subacute or chronic. The clinical features and laboratory findings of XPN resemble those of chronic pyelonephritis.

The symptoms are non-specific; the most common presenting sing of focal XPN is loin or flank pain, intermittent fever of unknown origin associated with anorexia, asthenia, a palpable flank or abdominal mass, and malaise with weight loss. Acute fever and flank tenderness are most common in the diffuse forms. Urinary symptoms such as dysuria are uncommon.

Laboratory results show a microcytic anaemia, an elevated erythrocyte sedimentation rate, leucocytosis, thrombocytosis and increased C-reactive protein in most patients.

Urinalysis may reveal pyuria, haematuria or proteinuria.

Positive urine cultures are predictive for XPN but are found in only 70% of all patients [3]. *Escherichia coli* and *Proteus mirabilis* are the most frequent organisms [6].

## Imaging features

Radiological findings diagnose and classify XPN into three stages depending on the extension of the inflammation, namely: stage I (nephric XPN), stage II (perinephric XPN) and stage III (paranephric XPN) [10, 11].

### Focal XPN

**Fig. 1:**
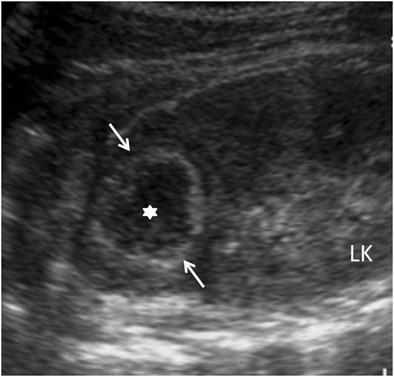
Focal XPN. A 13-year-old boy with left lumbar pain after playing football the day before. Longitudinal sonographic image shows focal hypoechoic mass (*asterisk*) with surrounding hyperechogenic rim (*white arrows*) in upper pole of left kidney

**Fig. 2:**
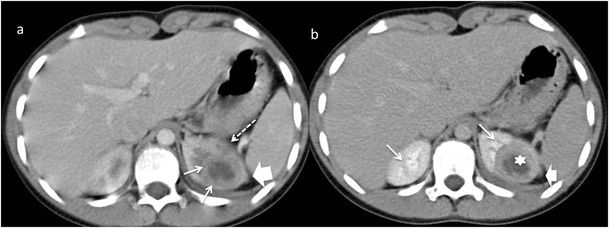
Focal XPN. Same patient as in Fig. 1. **a** Contrast-enhanced nephrogenic phase transverse CT image shows focal hypodense mass with rim enhancement (*white arrows*) and adjacent parenchymal destruction (*discontinuous white arrow*) in the cortex of the left kidney. Perirenal infiltration (*thick white arrow*) is present. **b** Contrast-enhanced excretory phase transverse CT image: the lesion is better delimited with thick rim enhancement and hypodense centre (*asterisk*). Working kidneys with contrast material excretion in the collecting system (*white arrows*) are shown. Perirenal infiltration (*thick white arrow*) is present

**Fig. 3:**
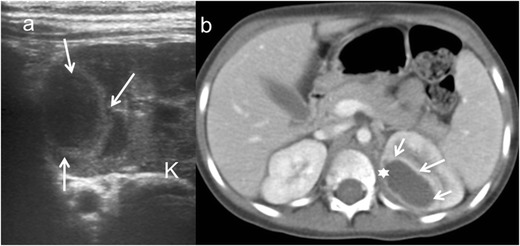
Renal abscess. A 5-year-old girl with fever. **a** Longitudinal sonographic image shows well-defined (*white arrows*) mass in upper pole of left kidney (*K*). **b** Contrast-enhanced nephrogenic phase transverse CT image shows hypodense lesion (*asterisk*) with rim enhancement (*white arrows*)

**Fig. 4:**
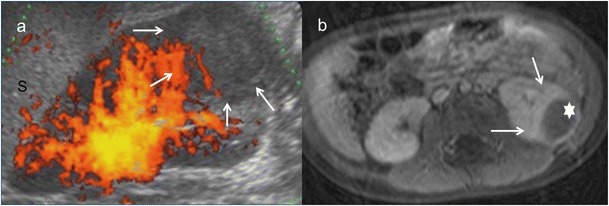
Wilms tumour. A 3-year-old girl with urinary infection. **a** Longitudinal power Doppler imaging shows an avascular area (*white arrows*) in lower pole of the left kidney (*S* spleen). **b** Contrast-enhanced fat-saturated T1-weighted axial image demonstrates hypointense mass (*white star*) with thick peripherical enhancement (*white arrows*). Surgery and histopathology proved a Wilms tumour

Focal XPN is a disease limited to a renal segment or one pole of a duplex system. US shows a localised hypoechoic mass, frequently without a stone obstructing the calyx (Fig. 1) [5, 12]. The kidney appears otherwise normal. CT demonstrates a well-defined localised intrarenal mass with water-like attenuation. There may also be a rim enhancement attributed to granulation tissue or compressed renal parenchyma. Perirenal extension may exist (Fig. 2). These findings are virtually impossible to differentiate from renal abscess (Fig. 3) or neoplasm (Fig. 4) [13, 14]. There are few data about MRI findings in the focal form. The lesion is isointense with the renal parenchyma on T1-weighted imaging and has slightly low signal intensity on T2-weighted. These findings suggest a fluid with a very high protein content. MRI, and especially the T2 sequences, are useful with the absence of hyperintensity in the differentiation of XPN from tumoural masses. The different signal intensity of the solid component of XPN on T1-weighted images, compared to the renal parenchyma, depends on the amount of xanthoma cells occurring in the lesion [1, 3, 15, 16].

### Diffuse XPN

**Fig. 5:**
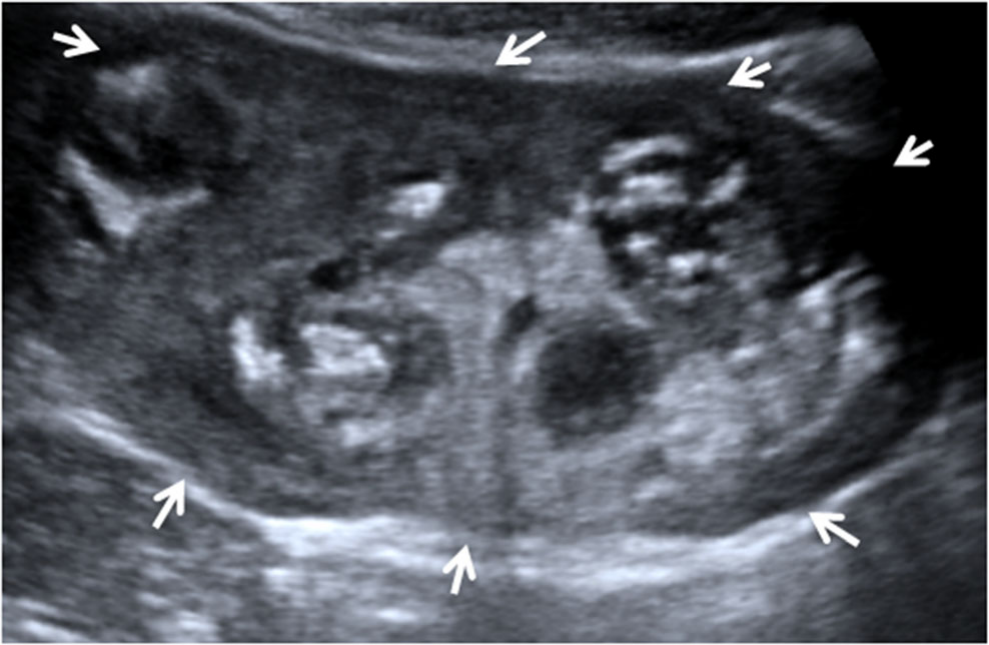
Diffuse XPN. Longitudinal sonogram image shows a large kidney maintaining reniform shape with heterogeneous content (*arrows*)

**Fig. 6:**
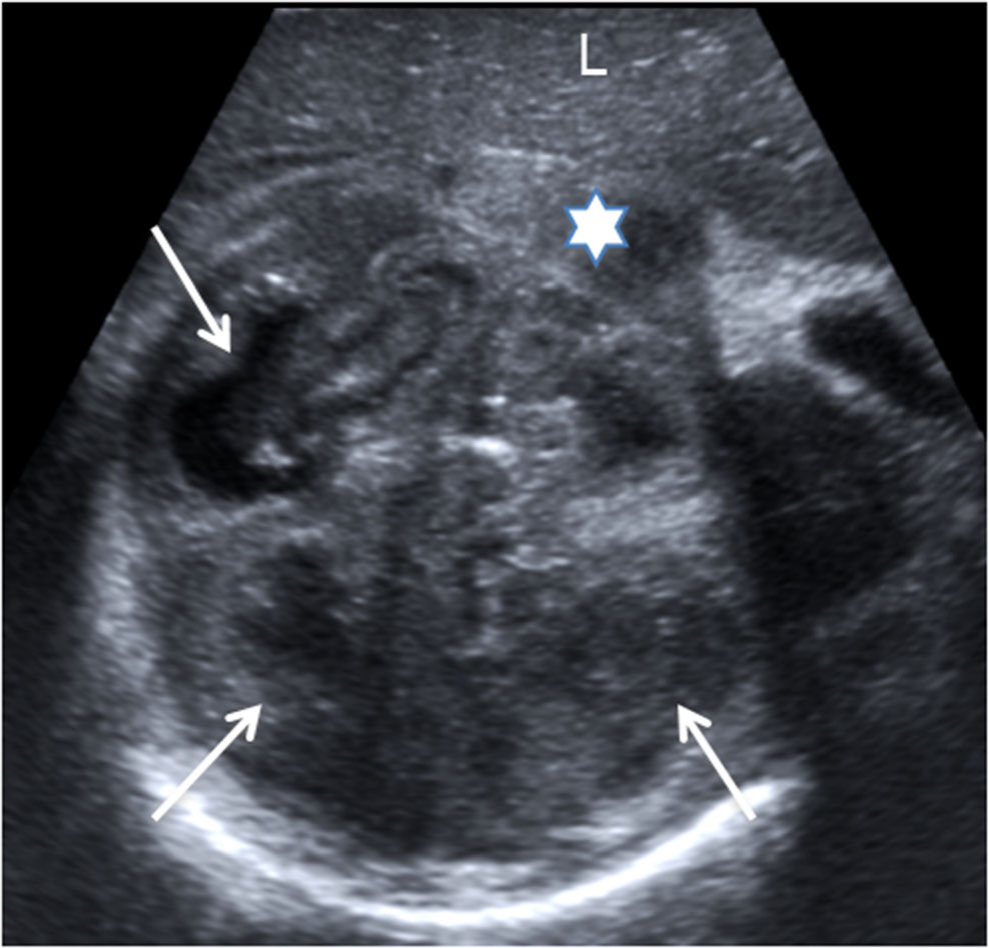
Diffuse XPN. Transverse sonogram image shows the right kidney parenchyma replaced with multiple hypoechoic masses (*white arrows*). Perinephric fluid collection is present (*star*). Liver (*L*)

**Fig. 7:**
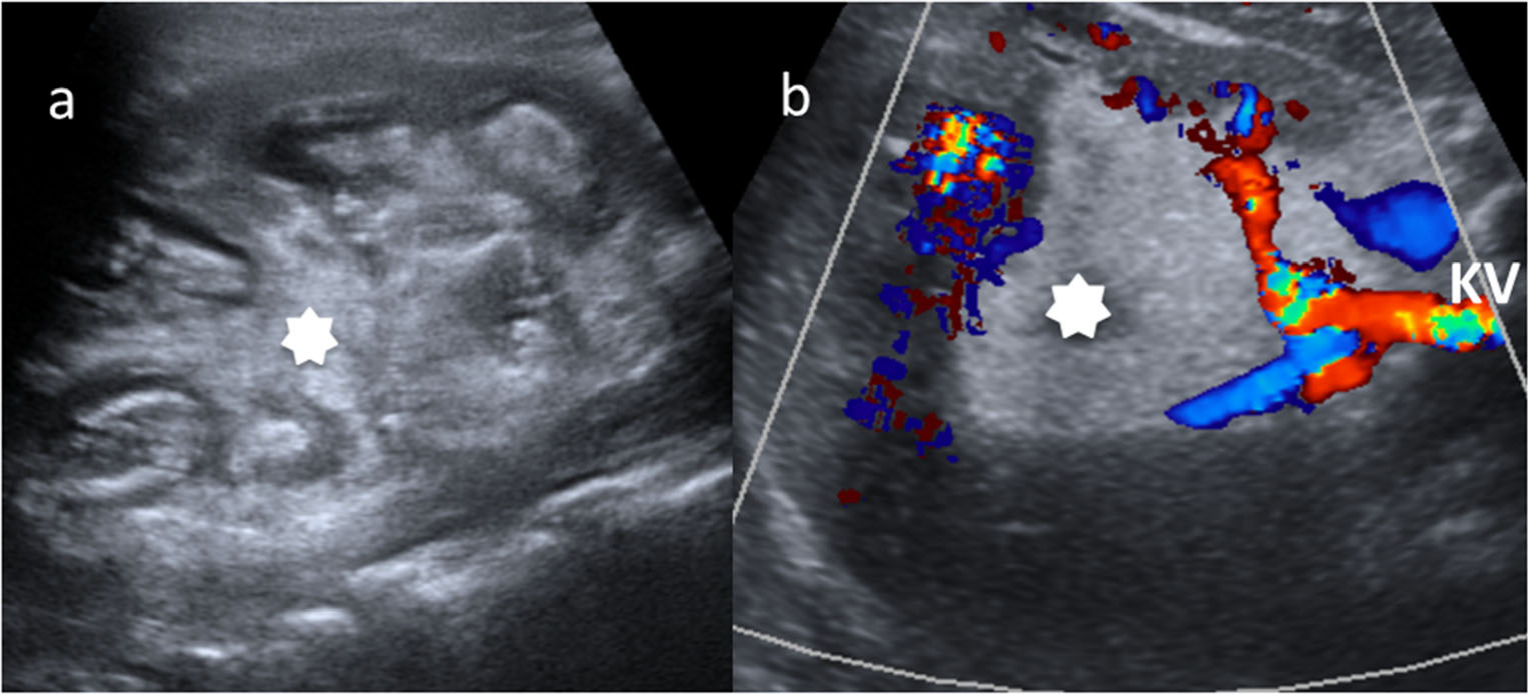
Diffuse XPN in left kidney. **a** Longitudinal ultrasound shows important inflammatory exudate and fatty proliferation (*white asterisk*) inside the kidney. **b** Transverse Doppler ultrasound shows kidney vessels (*KV*) passing through the inflammatory and fatty proliferation (*white asterisk*)

**Fig. 8:**
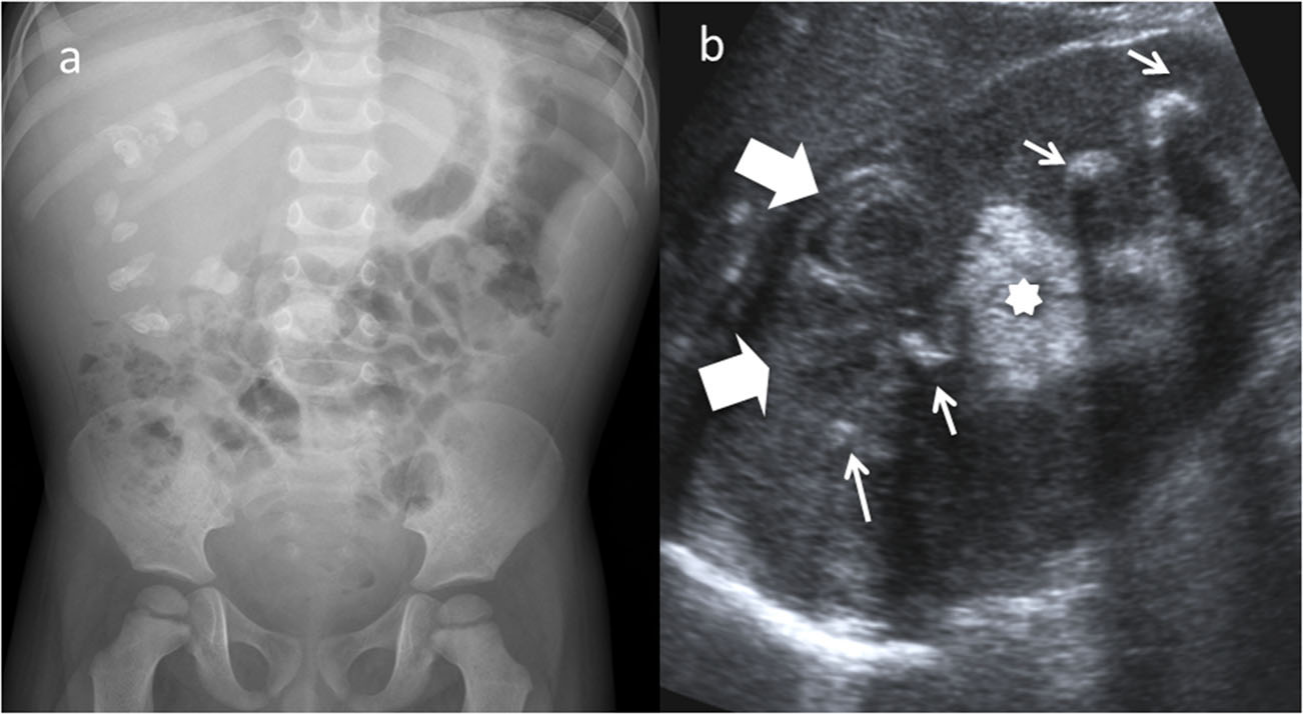
Diffuse XPN. **a** Plain X-ray of the abdomen showing multiple calculus in right kidney. **b** Longitudinal ultrasound shows calculus (*narrow white arrows*) in a large kidney with peripheric hypoechoic areas (*thick white arrows*) and inflammatory exudate (*asterisk*)

**Fig. 9:**
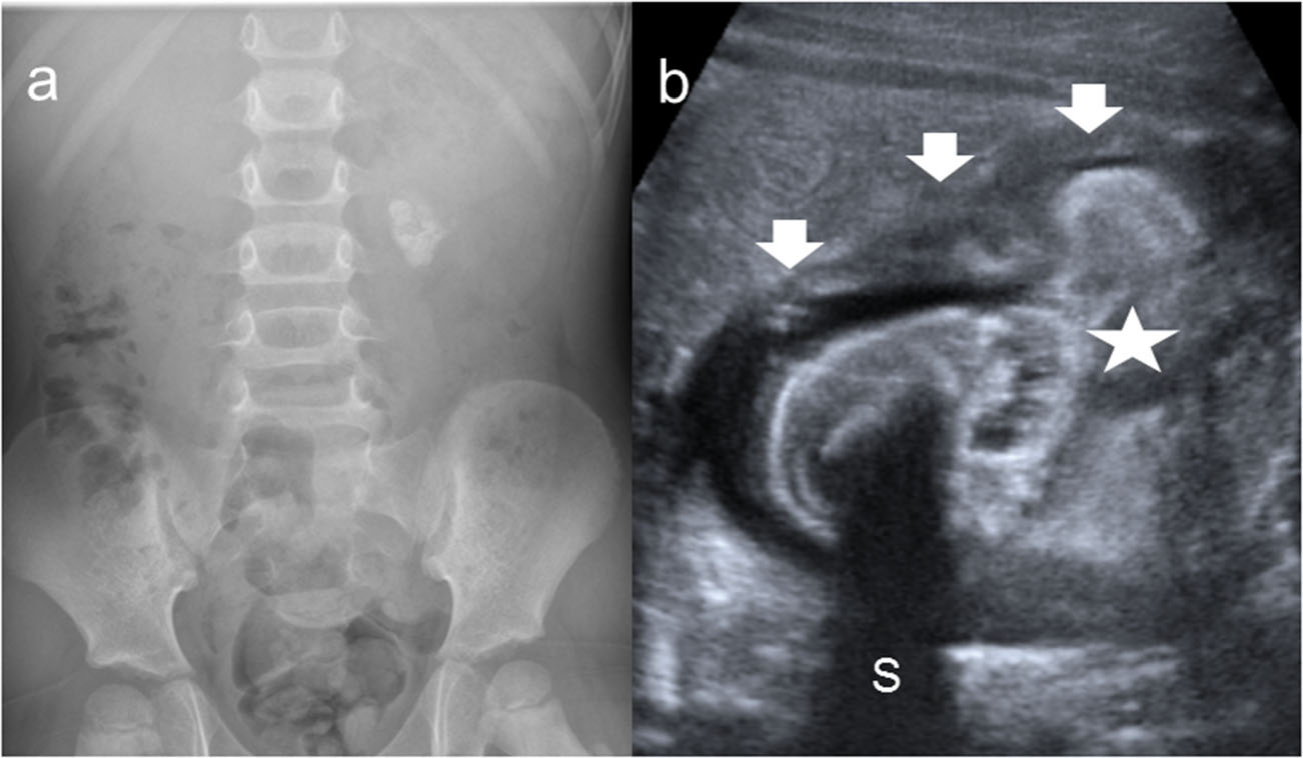
Diffuse XPN. **a** Plain X-ray of the abdomen showing a left staghorn calculus. **b** Transverse sonogram of the left kidney presents a stone with acoustic shadow (*S*) in a pelvis with important inflammatory and fatty component (*star*). Loss of parenchyma (*thick arrows*) is significant

In diffuse XPN, US shows an enlargement of the entire kidney although the reniform shape is maintained (Fig. 5). Multiple hypoechoic areas represent calyceal dilatation and parenchymal destruction. Depending on the extension, perinephritic fluid collection is present (Fig. 6). Fatty infiltration can be seen invading and expanding the kidney (Fig. 7). Lithiasis appears in up to 83% of XPN cases, calculus being from one-third to half staghorn (Figs. 8 and 9) [17].

**Fig. 10:**
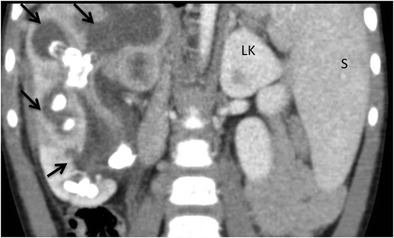
Diffuse XPN. Contrast-enhanced nephrogenic phase coronal reconstruction CT shows right kidney with multiple low attenuation round masses (*black arrows*) corresponding to either dilated calyces or focal areas of parenchymal destruction with numerous calculus in calyces. *LK* left kidney, *S* spleen

**Fig. 11:**
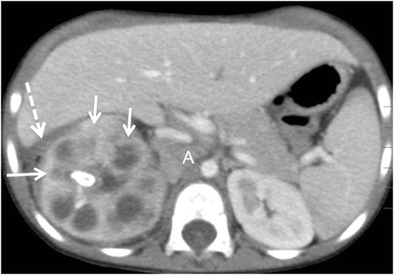
Diffuse XPN. Contrast-enhanced nephrogenic phase transverse CT shows enlarged right kidney with hypodense areas with peripheral enhancement (*white arrows*), a stone in the pelvis and extrarenal extension (*discontinuous arrows*). Adenopathy (*A*) displacing cava vein is present

**Fig. 12:**
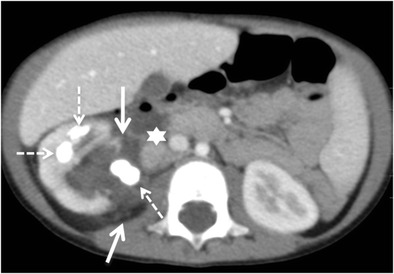
Diffuse XPN. Contrast-enhanced nephrogenic phase transverse CT shows multiple lithiasis in pelvis and calyces (*discontinuous arrows*), extrarenal extension (*white arrows*) and presence of adenopathy (*star*)

**Fig. 13:**
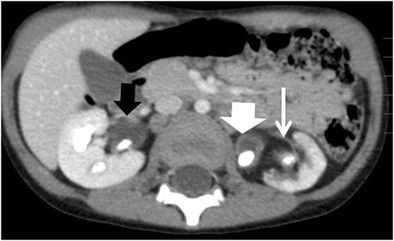
Diffuse XPN. Contrast-enhanced nephrogenic phase transverse CT shows bilateral kidney lithiasis. Right kidney presents a dilated pelvis with a stone inside, while left kidney shows a stone in the pelvis with fatty proliferation (*thin white arrow*) and an obstructive stone inside a dilated ureter (*thick white arrow*)

**Fig. 14:**
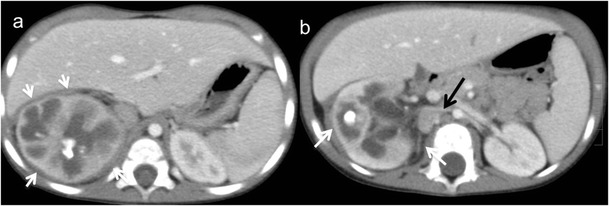
Diffuse XPN. **a** and **b** Contrast-enhanced nephrogenic phase transverse CT shows the massively enlarged affected kidney with inflammatory infiltration of perirenal fat (*white arrows*) and presence of adenopathy (*black arrow*)

**Fig. 15:**
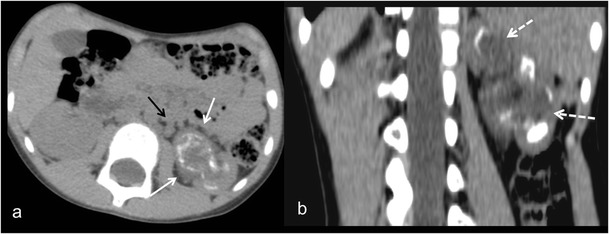
Atypical diffuse XPN. **a** Transverse and **b** coronal reconstructions CT show an atrophied kidney with multiple lithiasis, dystrophic calcifications in low attenuation areas of parenchyma destruction (*discontinuous arrows*), perirenal extension (*white arrows*) and retroperitoneal adenopathies (*black arrows*)

CT is the cornerstone of diagnostic imaging for diffuse XPN. The typical CT appearance is of global renal enlargement with multiple low-attenuation rounded areas replacing the renal parenchyma (Fig. 10). These represent either dilated calyces or focal areas of destruction filled with pus or debris and have been described as the “bear paw sign”. On CT after intravenous contrast, the walls of these cavities demonstrate enhancement, due to vascular granulation tissue (Fig. 11). The renal pelvis is usually contracted around a central calculus, although calculus can be multiple and diffuse inside different calyces (Fig. 12). The presence of lipomatous component helps to diagnose XPN (Fig. 13). CT identifies extension of extrarenal disease to involve the perinephric and paranephric spaces, the posterior abdominal wall or psoas muscle (Fig. 14) [18, 19]. Adenopathies are frequently present. In adults, a study reports that 87% of patients who had undergone CT were well diagnosed preoperatively [20]. However, less common CT features can be seen: absence of calculi, important pelvic dilatation or renal atrophy (Fig. 15) [14, 21].

MRI findings in diffuse XPN are variable, probably because the signal intensity of the solid component of XPN on T1-weighted images depends on the amount of xanthoma cells involved in the granulomatous process. The signal intensity on T2-weighted images is isointense compared to the normal kidney. The content of the cavities is hypointense on T1-weighted images and hyperintense on T2-weighted with fluid-fluid levels. Perirenal infiltration is hypointense on both T1 and T2 sequences due to the presence of thick fibrinous exudate. Extensive sinus replacement lipomatosis is easily recognised with MRI. After the administration of a contrast medium (gadolinium-DTPA), the rim enhancements of the borders of the cavities and delineates perirenal inflammatory extension with bright and thickening of perirenal fascia. This finding is important for surgical planning, and MRI visualises it better than CT. Progressively, MRI will replace CT as the method of choice for the diagnosis of XPN [22].

**Fig. 16:**
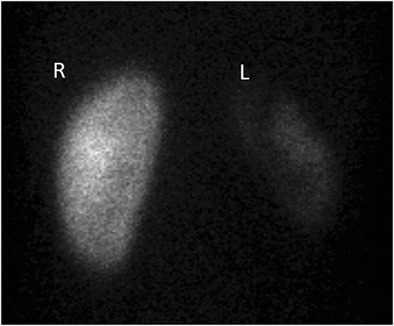
Diffuse XPN. The same patient as in Fig. 15. DMSA scan shows good uptake in normal right kidney (*R*) and poor renal uptake in left kidney (*L*)

Non-function or very low renal function is seen by dimercaptosuccinic acid (DMSA) scintigraphy (Fig. 16) [23].

## Management

The management of XPN differs between focal and diffuse form.

### Focal XPN

Owing to the difficult differential diagnosis of focal XPN with renal tumours (Wilms’s tumour), it is often necessary to perform either image-guided biopsy or intraoperative biopsy to avoid unnecessary nephrectomy [1, 24, 25]. In focal XPN, treatment modes are partial nephrectomy, drainage of XPN abscess, or broad-spectrum antibiotic. There have been case reports of successful medical management of focal XPN [26], but it is unusual because of uncertain prognostic.

### Diffuse XPN

Clinical and imaging features are suggestive of diffuse XPN and biopsy is not required before nephrectomy [20], which remains as the standard of care for diffuse disease. Nevertheless, the indication for nephrectomy is not always straightforward. There is no published evidence suggesting whether differential function can be used to guide the treatment of diffuse XPN. Differential function of near 20% is used as a recommendation for nephrectomy [17]. Up to 80.5% of the affected kidneys are non-functioning [9].

**Fig. 17:**
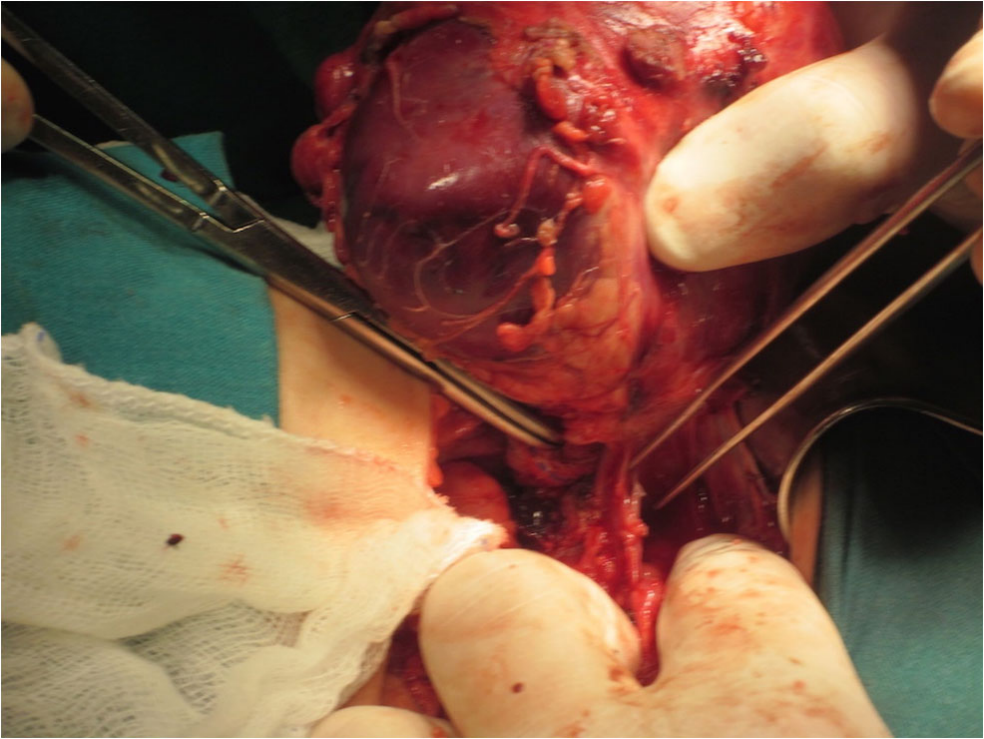
Surgical procedure. Presence of multiples adherences and adenopathies around the kidney during the nephrectomy

Surgery of diffuse XPN can be difficult as a result of inflammatory processes extending beyond the kidney (Fig. 17), and although laparoscopic procedure is the first option [27], sometimes it must be changed to lumbotomy or anterolateral transperitoneal approach. Cutaneous fistula, bowel fistula and delayed wound healing have been reported as complications; consequently, in order to improve the surgical results, it is recommended to drain the perirenal and/or renal abscess prior to surgery [3, 28].

The management of bilateral XPN is difficult due to the risk of permanent renal dysfunction. Partial nephrectomy is advocated but more recently conservative management with antibiotic therapy has been proposed [1, 9].

The diffuse type of XPN is associated with a worse prognosis compared to the focal type, although no recurrence of XPN in the healthy contralateral kidney has been reported.

## Histopathological findings

The final diagnosis of XPN is histopathological.

The initial description of the pathological features of XPN was published in 1916 by Schlagenhaufer [29], but the first paediatric cases were not described until 1963 by Avnet et al. [30] and Friedenberg and Spjut [31].

**Fig. 18:**
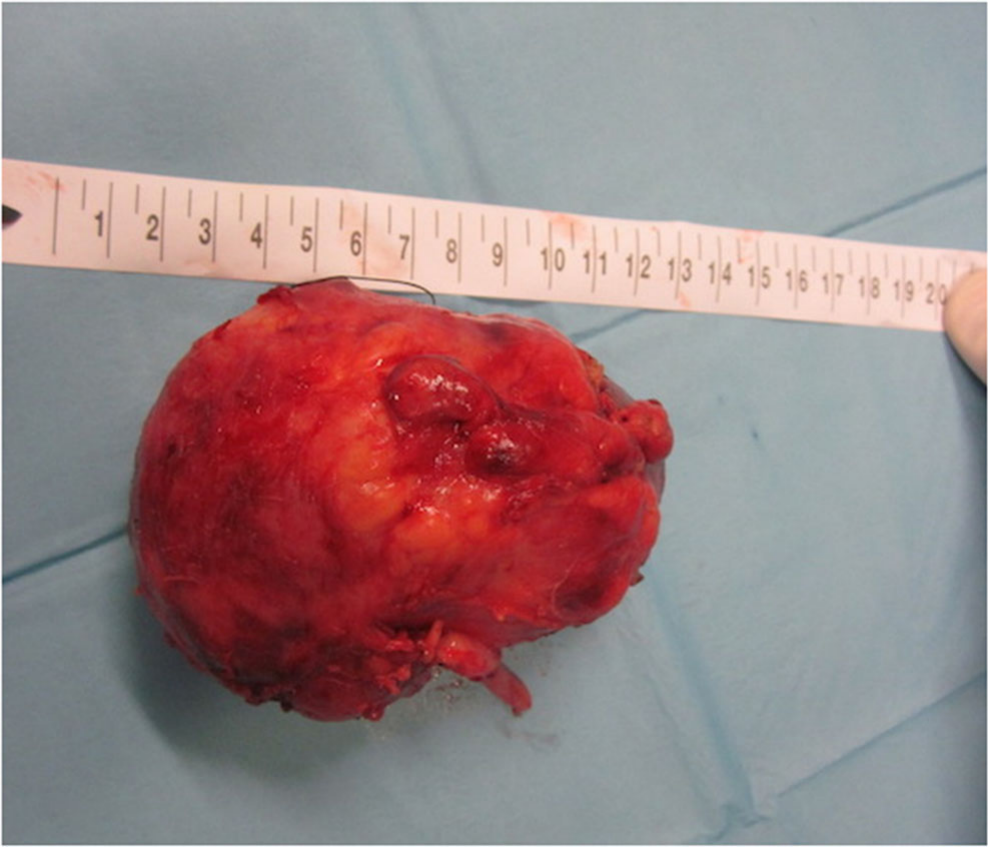
Gross specimen photograph shows a large kidney maintaining reniform shape

**Fig. 19:**
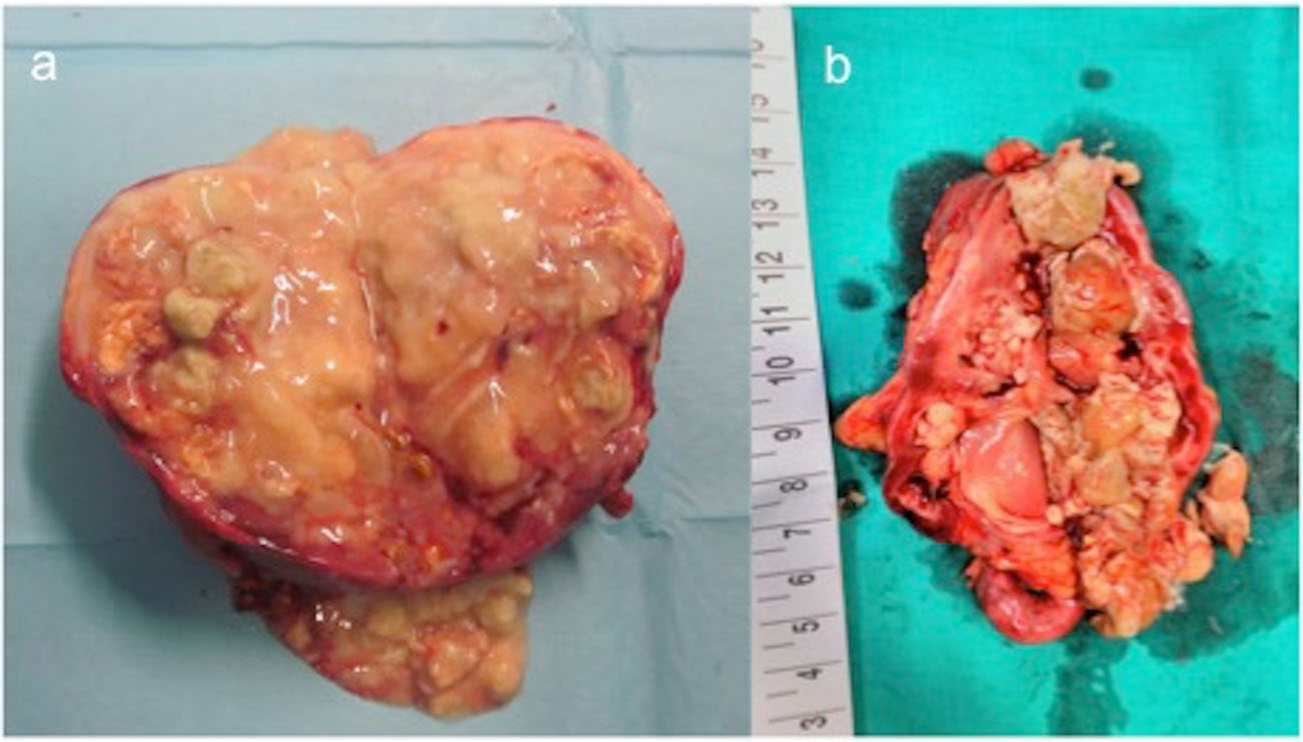
Gross specimen photograph of the bivalved kidneys demonstrate **a** multiple large, necrotic spaces filled with purulent material and surrounded by nodular yellow masses and **b** an enlarged kidney with destruction of normal renal parenchyma. Yellow pus and debris fill calyces and replace renal parenchyma

**Fig. 20:**
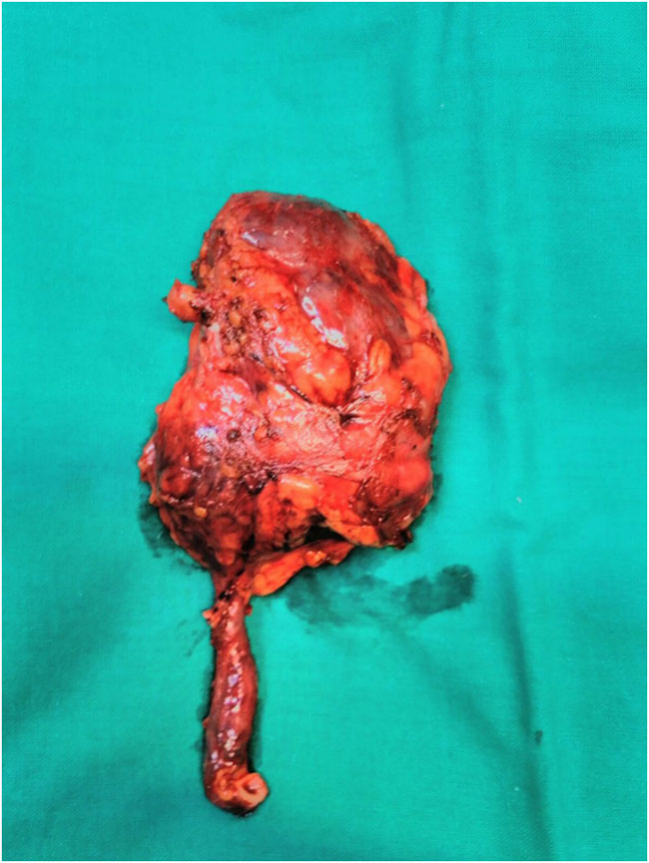
Gross specimen shows a large kidney with ureteral pyelic junction stenosis favouring the development of the XPN

After nephrectomy by diffuse XPN, gross examination shows an enlarged kidney with a thickened capsule (Fig. 18) and a marked loss of renal parenchymal replaced by yellow fatty nodules with or without central necrosis. The renal pelvis and calyces are dilated and filled with stones, debris or pus (Fig. 19). Sometimes, a structural anomaly such as a narrow ureteropelvic junction may be present (Fig. 20) [3].

**Fig. 21:**
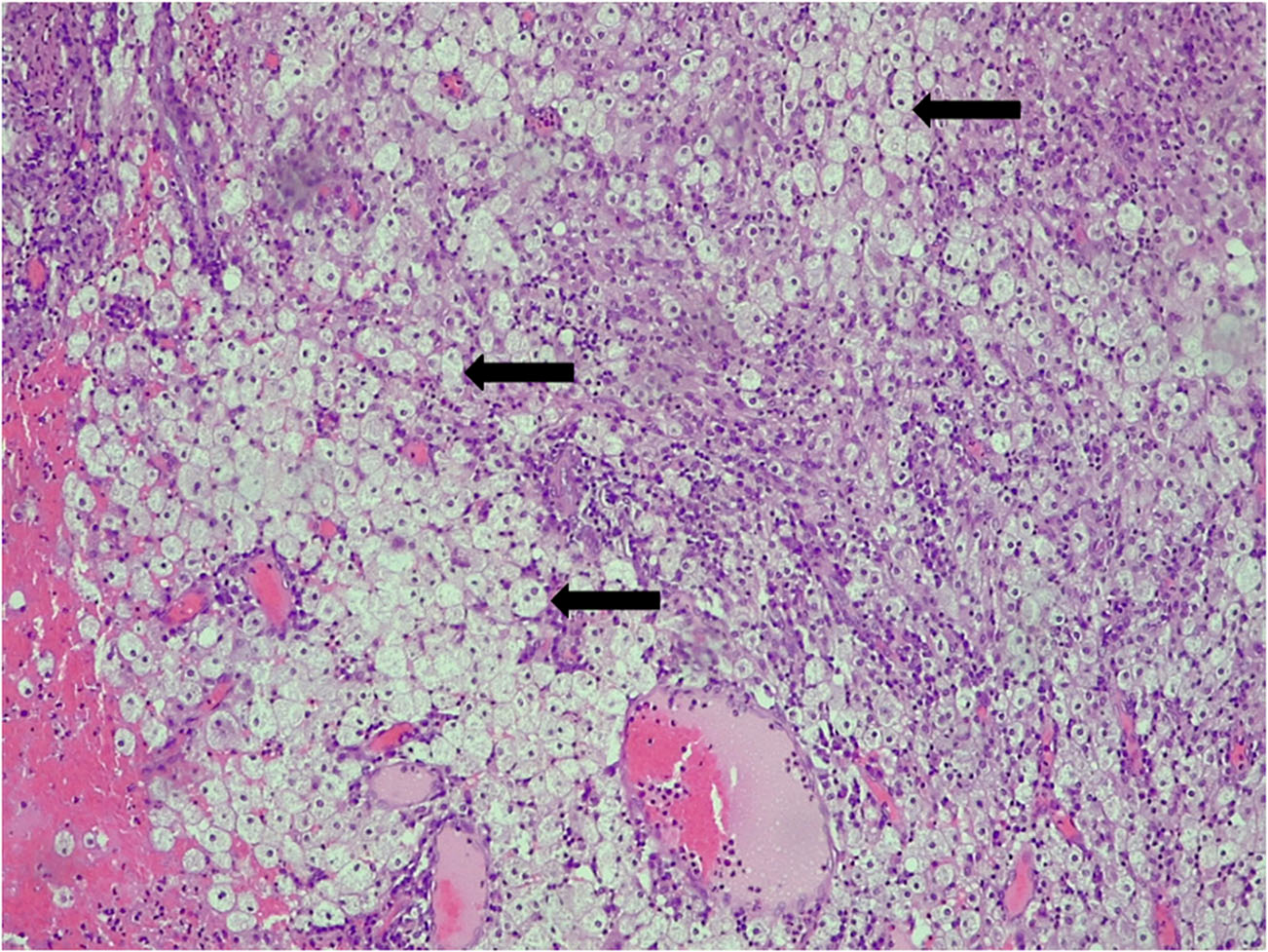
Micrograph of a histological section (×10) haematoxylin and eosin stain showing typical foamy macrophages (*black arrows*)

Diffuse and focal XPN microscopic findings include a mixed acute and chronic inflammatory cell infiltrate with giant cells and lipid-laden macrophages (foam cells) (Fig. 21). Discrete lymphoid follicles, granulation tissue, intensive fibrosis and hyalinised glomeruli sclerosis may identify [5, 11, 16, 32].

## Conclusions

XPN in children is a rare condition and preoperative diagnosis, especially of the focal type, remains challenging. Increasingly sensitive radiological methods, such as US, CT and MRI in combination with clinical suspicion enable the diagnosis. There are no clear guidelines on the management of XPN, thus conservative and surgical treatments should be considered for each individual case. The final diagnosis is histopathological.

## Abbreviation

XPN: Xanthogranulomatous pyelonephritis
